# Notes from Foreign Societies

**Published:** 1882-04

**Authors:** 


### NOTES FROM FOREIGN SOCIETIES.

PHARYNGEAL TUBERCULOSIS.—Dr. Millard presented to the Société Médicale des Hopitaux, in December last, anatomical specimens from a young man who recently died of miliary tuberculosis at the age of 17. Patient died of starvation after a month's agony; the sufferings caused by the ingestion of food were such that natural deglutition and the introduction of Fancher's syphon had been impossible. Dr. Millard vainly tried to feed the patient through a flexible sound passed through the nostril. Patient died without any perceptible sound in chest other than a weakening of the respiratory murmur.

At the autopsy there were deep lesions in the velum palati, the pharynx and the epiglottis. The tongue and larynx were sound. The intestines were covered with tubercular granulations and ulcerations. The lungs contained recent tubercular lesions which accounted for the absence of pulmonary râles during life. There were some miliary tubercles in the kidneys, but none in the genital organs. This was a striking type of generalized miliary tuberculosis, having terminated in pharyngeal tuberculosis.—*La France Médicale.*

DILATATION AND SUPPURATION OF THE FALLOPIAN TUBES.—At a meeting of the Pathological Society of London, December 6, 1881, Mr. Lawson Tait related some cases of the conditions termed hydro-and pyo-salpinx. In the first case related there had been dysmenorrhœa for three years. On each side of the uterus was a hard tender mass. Abdominal section was performed, and the ovaries and appendages, which were inflamed and glued together, were removed. In the second case also there was intense dysmenorrhœa. The ovaries were enlarged, and the uterus was large and tender; the ovaries and Fallopian tubes were removed by abdominal section, and the patient made a good recovery. The third specimen was removed from a woman who for five years had been constantly ill; the ovaries and appendages were removed, and the patient made a speedy recovery. The fourth specimen was one met with in the course of an ovariotomy for cystoma. The nature of the cyst was obscure, but it seemed a dilated Fallopian tube. The fifth specimen was taken from a woman who had had an attack of pelvic inflammation, and for years afterwards suffered from dysmenorrhœa. A good recovery followed removal of the ovaries and appendages by abdominal section. The sixth specimen was from a young woman with dysmenorrhœa and menorrhagia. Removalof the ovaries was at first declined, but in August last the distended ovaries and appendages were removed. This patient also recovered rapidly and well. Another patient had suffered for many months from constant pain, aggravated at the menstrual period. In October last the ovaries and appendages were removed. The Fallopian tubes both contained a large quantity of pus. A good recovery ensued. In another case there was a clear history of pelvic peritonitis, followed by dysmenorrhœa. The distended Fallopian tubes and the ovaries were removed, and the patient made a good recovery. In another case there was a history of acute pelvic peritonitis, and subsequently dysmenorrhœa and menorrhagia became prominent symptoms. The Fallopian tubes, which were distended with purulent fluid, and the ovaries, which were large and soft, were removed. She also made a good recovery. Summing up the result of his experience, Mr. Tait said that a history pointing to an attack of pelvic suppuration, with the subsequent development of dysmenorrhœa, and frequently of menorrhagia, and of pain on intercourse was generally to be obtained in these cases. By physical examination, sausage-shaped tumors could be discovered behind or on each side of the uterus. The enlarged tubes were generally firmly attached to the uterus, and their removal was often a matter of considerable difficulty, so that the operations were often very tedious. He had now performed the operation twenty-two times, and all his patients had recovered from the operation, and had subsequently been free from any symptoms. He thought that all the cases owed their origin to pelvic peritonitis, with adhesion of the Fallopian tube to the ovary, and its consequent occlusion. In one other case, which was not operated on, death was due to rupture of the right Fallopian tube into the peritoneum; this set up severe peritonitis. The operation was followed by cessation of menstruation and by sterility.—*British Medical Journal.*

---

NUMBER OF PHYSICIANS.—According to calculations made by the Medical Academy of Paris, there are at present 189,000 doctors scattered over the world. Of these there are 65,000 in the United States; 26,000 in France; 32,000 in Germany and Austria; 35,000 in Great Britain and its colonies; 11,000 in Italy, and 5,000 in Spain. Putting aside pamphlets and memoirs innumerable, it is estimated that 120,000 works have been published on medical subjects. Of the writers, 2,800 are American; 2,600 French; 2,300 German and Austrian, and 2,100 English. —*Cincinnati Lancet and Clinic.*

